# 1-(4-Hy­droxy­phen­yl)-2-(2-oxidonaphthalen-1-yl)diazen-1-ium methanol hemisolvate

**DOI:** 10.1107/S1600536813012245

**Published:** 2013-05-11

**Authors:** Mohamed Amine Benaouida, Souheyla Chetioui, Salah Eddine Bouaoud

**Affiliations:** a Unité de Recherche de Chimie de l’Environnement et Moléculaire Structurale, (CHEMS), Faculté des Sciences Exactes, Département de Chimie, Université Constantine 1, 25000 Constantine, Algeria

## Abstract

In the title compound, C_16_H_12_N_2_O_2_·0.5CH_3_OH, the H atom of the –OH group has been transfered to the N atom in the azo group, forming a zwitterion. Hence, there is an intra­molecular N—H⋯O, rather than an O—H⋯N, hydrogen bond in the mol­ecule. The mol­ecule is almost planar, the dihedral angle between the benzene ring and the mean plane of the naphthalene ring system being 4.51 (6)°. In the crystal, mol­ecules are linked to and bridged by O—H⋯O hydrogen bonds involving the methanol mol­ecule, which is located about a twofold rotation axis, and hence half-occupied, forming zigzag chains along [001]. Mol­ecules are also linked *via* C—H⋯π and π–π inter­actions, the latter involving adjacent benzene and naphthalene rings and having a centroid–centroid distance of 3.6616 (13) Å, forming a three-dimensional network.

## Related literature

For azo compounds in the fields of dyes, pigments and advanced materials, see: Lee *et al.* (2004 ▶); Oueslati *et al.* (2004 ▶). For the synthesis of azo compounds, see: Wang *et al.* (2003 ▶). For the structures of related compounds, see: Jin *et al.* (2008 ▶); Xu *et al.* (2010 ▶).
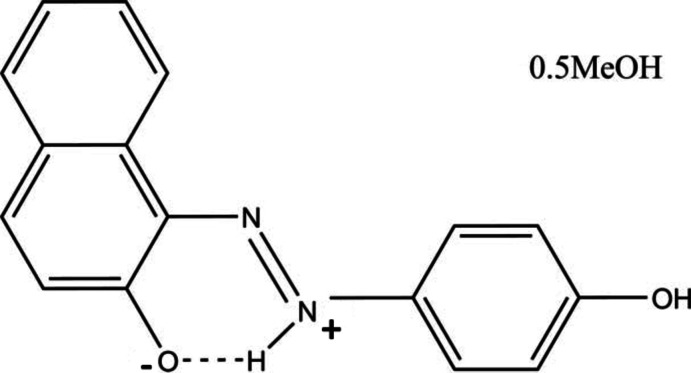


## Experimental

### 

#### Crystal data

C_16_H_12_N_2_O_2_·0.5CH_4_O
*M* *_r_* = 280.30
Monoclinic, 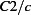

*a* = 26.942 (7) Å
*b* = 6.3479 (17) Å
*c* = 17.579 (5) Å
β = 113.985 (4)°
*V* = 2746.8 (13) Å^3^
*Z* = 8
Mo *K*α radiation
μ = 0.09 mm^−1^
*T* = 293 K
0.26 × 0.06 × 0.05 mm

#### Data collection

Bruker APEXII CCD diffractometer
12992 measured reflections
4212 independent reflections
2286 reflections with *I* > 2σ(*I*)
*R*_int_ = 0.032

#### Refinement

*R*[*F* ^2^ > 2σ(*F* ^2^)] = 0.052
*wR*(*F* ^2^) = 0.154
*S* = 1.03
4212 reflections
202 parameters
H atoms treated by a mixture of independent and constrained refinement
Δρ_max_ = 0.18 e Å^−3^
Δρ_min_ = −0.18 e Å^−3^

### 

Data collection: *APEX2* (Bruker, 2006 ▶); cell refinement: *SAINT* (Bruker, 2006 ▶); data reduction: *SAINT*; program(s) used to solve structure: *SHELXS97* (Sheldrick, 2008 ▶); program(s) used to refine structure: *SHELXL97* (Sheldrick, 2008 ▶); molecular graphics: *PLATON* (Spek, 2009 ▶); software used to prepare material for publication: *WinGX* publication routines (Farrugia, 2012 ▶).

## Supplementary Material

Crystal structure: contains datablock(s) I, global. DOI: 10.1107/S1600536813012245/su2594sup1.cif

Structure factors: contains datablock(s) I. DOI: 10.1107/S1600536813012245/su2594Isup2.hkl

Supplementary material file. DOI: 10.1107/S1600536813012245/su2594Isup3.cml

Additional supplementary materials:  crystallographic information; 3D view; checkCIF report

## Figures and Tables

**Table 1:** Hydrogen-bond geometry (Å, °) *Cg*2 and *Cg*3 are the centroids of the C5–C10 and C11–C16 rings, respectively.

*D*—H⋯*A*	*D*—H	H⋯*A*	*D*⋯*A*	*D*—H⋯*A*
O1*S*—H1*O*⋯O1^i^	0.92	1.92	2.832 (3)	171
N2—H2*A*⋯O1	0.86	1.84	2.540 (2)	137
O2—H2*B*⋯O1*S*	0.82	2.03	2.841 (3)	172
O2—H2*B*⋯O1*S* ^ii^	0.82	1.97	2.690 (3)	146
C1*S*—H1*SA*⋯*Cg*3^iii^	1.11 (6)	2.58 (5)	3.555 (2)	147 (4)
C7—H7*A*⋯*Cg*2^iv^	0.93	2.73	3.521 (2)	144

Symmetry codes: (i) 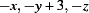; (ii) 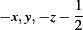; (iii) 
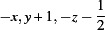
; (iv) 
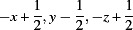
.

**Fig. 1.**
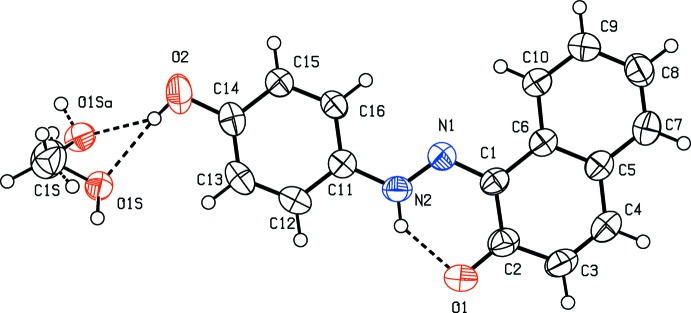
View of the molecular structure of the title compound, with atom labelling. Displacement ellipsoids are drawn at the 50% probability level.

**Fig. 2.**
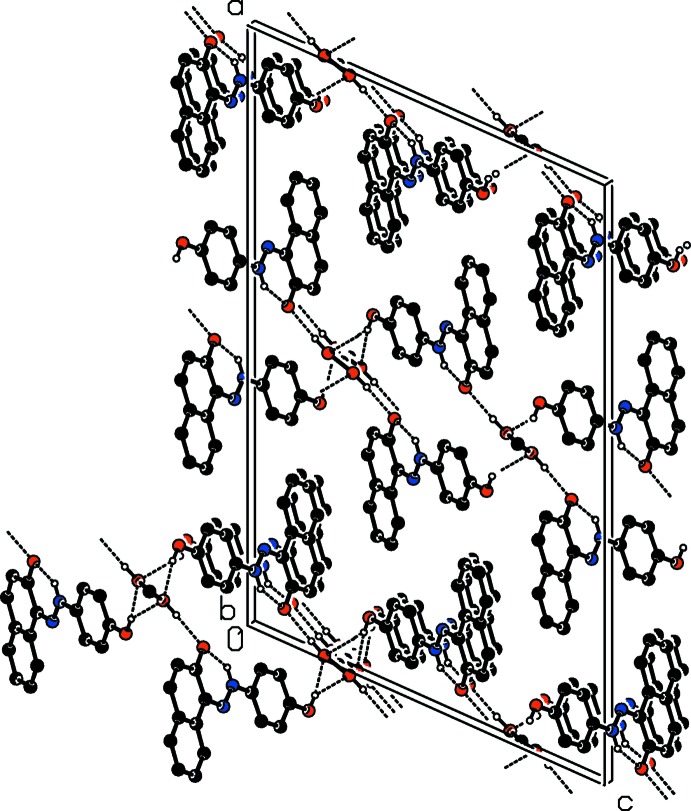
A view along the axis of the crystal packing of the title compound. The various hydrogen bonds are shown as dashed lines (see Table 1 for details).

## supplementary crystallographic information

### Comment

Azo compounds are very important in the fields of dyes, pigments and advanced materials (Lee *et al.*, 2004; Oueslati *et al.*, 2004). Characterized by the azo linkage (–N=N–). Many azo compounds have been synthesized by the diazotization and a diazo coupling reaction (Wang *et al.*, 2003), followed by a coupling reaction with 2-naphthol. This entails an electrophilic substitution reaction where an aryl diazonium cation attacks another aryl ring. Since diazonium salts are often unstable near room temperature, the azo coupling reactions are typically conducted near ice temperatures. The pH of the solution is quite important; it must be mildly acidic or neutral, since no reaction takes place if the pH is too low. We report herein on the crystal structure of the title compound, obtained through the diazotization of 4-hydroxyaniline followed by a coupling reaction with 2-naphthol. The synthesis and structure of a 4-methylaniline (Wang *et al.*, 2003) and an aniline (Jin *et al.*, 2008; Xu *et al.*, 2010) analogue of the title compound have been described.

In the title molecule, Fig. 1, the bond lengths and angles are within normal ranges. Interestingly, the hydrogen atom of the OH group has been transfered to the N2 atom in the azo group to form a dipolar ion; the difference Fourier map indicated that the hydrogen atom site location is closer to the N atom of the azo group. Hence, there is an intramolecular N—H···O, rather than an O-H···N, hydrogen bond in the molecule (Fig. 1 and Table 1). The molecule is relatively plane, with mean plane of the naphthalene ring system (C1-C10) oriented at a dihedral angle of 4.51 (6) ° with respect to the benzene ring (C11-C16).

In the crystal, molecules are bridged by O-H···O hydrogen bonds, involving the methanol molecule which is located about a twofold rotation axis, forming chains along [001]; see Table 1 and Fig. 2. Molecules are also linked via C-H···π (Table 1) and π–π interactions interactions, forming a three-dimensional structure. The latter interactions involve adjacent benzene and naphthalene rings [Cg1···Cg3^i^ = 3.6616 (13) Å; Cg1 is the centroid of ring C1-C6; Cg3 is the centroid of ring C11-C16; symmetry code: (i) x, y-1, z].

### Experimental

The title compound was prepared by the method of (Wang *et al.*, 2003) for similar aromatic azo–compounds. Red prismatic crystals of the title compound were obtained by slow evaporation of a solution in methanol.

### Refinement

The hydrogen atom of the OH group was located in a difference Fourier map and found to be near to the N atom, N2, of the azo group. In the final cycles of refinement it was included in a calculated position and treated as a riding atom: N-H = 0.86 Å with *U*_iso_(H) = 1.2U_eq_(N). The C-bound H atoms were positioned geometrically and refined as riding: C-H = 0.93 Å with *U*_iso_(H) = 1.2U_eq_(C). The disordered methanol solvent OH and CH_3_ H atoms were located in a difference Fourier map and refined as riding atoms with *U*_iso_(H) = 1.5U_eq_(O,C). Two reflections (2 0 0 and 1 1 3) likely affected by the beamstop were omitted in the final cycles of refinement.

### Crystal data

**Table:**

C_16_H_12_N_2_O_2_·0.5CH_4_O	*F*(000) = 1176
*M**_r_* = 280.30	Least-squares treatment of 25 SET4 setting angles.
Monoclinic, *C*2/*c*	*D*_x_ = 1.356 Mg m^−^^3^
Hall symbol: -C 2yc	Mo *K*α radiation, λ = 0.71073 Å
*a* = 26.942 (7) Å	Cell parameters from 1832 reflections
*b* = 6.3479 (17) Å	θ = 2.4–25.7°
*c* = 17.579 (5) Å	µ = 0.09 mm^−^^1^
β = 113.985 (4)°	*T* = 293 K
*V* = 2746.8 (13) Å^3^	Prismatic, red
*Z* = 8	0.26 × 0.06 × 0.05 mm

### Data collection

**Table:**

Bruker APEXII CCD diffractometer	2286 reflections with *I* > 2σ(*I*)
Radiation source: fine-focus sealed tube	*R*_int_ = 0.032
Graphite monochromator	θ_max_ = 30.7°, θ_min_ = 2.4°
phi and ω scans	*h* = −38→37
12992 measured reflections	*k* = −8→9
4212 independent reflections	*l* = −24→24

### Refinement

**Table:**

Refinement on *F*^2^	Primary atom site location: structure-invariant direct methods
Least-squares matrix: full	Secondary atom site location: difference Fourier map
*R*[*F*^2^ > 2σ(*F*^2^)] = 0.052	Hydrogen site location: inferred from neighbouring sites
*wR*(*F*^2^) = 0.154	H atoms treated by a mixture of independent and constrained refinement
*S* = 1.03	*w* = 1/[σ^2^(*F*_o_^2^) + (0.0652*P*)^2^ + 0.3604*P*] where *P* = (*F*_o_^2^ + 2*F*_c_^2^)/3
4212 reflections	(Δ/σ)_max_ = 0.001
202 parameters	Δρ_max_ = 0.18 e Å^−^^3^
0 restraints	Δρ_min_ = −0.18 e Å^−^^3^

### Special details

**Table:**

Geometry. Bond distances, angles etc. have been calculated using the rounded fractional coordinates. All su's are estimated from the variances of the (full) variance-covariance matrix. The cell esds are taken into account in the estimation of distances, angles and torsion angles
Refinement. Refinement of F^2^ against ALL reflections. The weighted R-factor wR and goodness of fit S are based on F^2^, conventional R-factors R are based on F, with F set to zero for negative F^2^. The threshold expression of F^2^ > 2sigma(F^2^) is used only for calculating R-factors(gt) etc. and is not relevant to the choice of reflections for refinement. R-factors based on F^2^ are statistically about twice as large as those based on F, and R- factors based on ALL data will be even larger.

### Fractional atomic coordinates and isotropic or equivalent isotropic displacement parameters (Å^2^)

**Table:**

	*x*	*y*	*z*	*U*_iso_*/*U*_eq_	Occ. (<1)
O1	0.05411 (5)	0.9010 (2)	0.10115 (8)	0.0693 (5)	
O2	0.09325 (5)	1.7942 (2)	−0.17549 (9)	0.0775 (5)	
N1	0.13875 (5)	0.98315 (19)	0.04654 (7)	0.0426 (4)	
N2	0.09869 (5)	1.1128 (2)	0.02339 (7)	0.0454 (4)	
C1	0.13793 (5)	0.8158 (2)	0.09375 (8)	0.0408 (4)	
C2	0.09520 (6)	0.7749 (3)	0.12016 (10)	0.0516 (5)	
C3	0.09810 (7)	0.5873 (3)	0.16635 (10)	0.0563 (6)	
C4	0.13956 (6)	0.4517 (3)	0.18484 (9)	0.0510 (5)	
C5	0.18354 (6)	0.4878 (2)	0.16088 (8)	0.0439 (5)	
C6	0.18351 (5)	0.6722 (2)	0.11622 (8)	0.0402 (4)	
C7	0.22698 (6)	0.3453 (3)	0.18175 (9)	0.0529 (5)	
C8	0.26920 (7)	0.3855 (3)	0.16014 (10)	0.0590 (6)	
C9	0.26971 (6)	0.5681 (3)	0.11681 (10)	0.0565 (6)	
C10	0.22765 (6)	0.7090 (3)	0.09506 (9)	0.0482 (5)	
C11	0.09846 (5)	1.2857 (2)	−0.02644 (8)	0.0421 (4)	
C12	0.05513 (6)	1.4243 (3)	−0.04920 (10)	0.0505 (5)	
C13	0.05257 (6)	1.5962 (3)	−0.09868 (10)	0.0555 (5)	
C14	0.09319 (6)	1.6298 (2)	−0.12578 (9)	0.0507 (5)	
C15	0.13698 (6)	1.4922 (2)	−0.10218 (9)	0.0497 (5)	
C16	0.13961 (6)	1.3214 (2)	−0.05285 (9)	0.0467 (5)	
O1S	−0.00722 (9)	2.0049 (3)	−0.21505 (14)	0.0490 (7)	0.500
C1S	0.00000	2.1846 (5)	−0.25000	0.0803 (14)	
H2A	0.07230	1.09380	0.03830	0.0540*	
H2B	0.06390	1.85490	−0.19190	0.1160*	
H3A	0.07060	0.55830	0.18400	0.0680*	
H4A	0.13970	0.32990	0.21430	0.0610*	
H7A	0.22690	0.22250	0.21060	0.0630*	
H8A	0.29780	0.29040	0.17440	0.0710*	
H9A	0.29880	0.59470	0.10250	0.0680*	
H10A	0.22840	0.83040	0.06590	0.0580*	
H12A	0.02760	1.40130	−0.03110	0.0610*	
H13A	0.02350	1.68920	−0.11370	0.0670*	
H15A	0.16470	1.51600	−0.11990	0.0600*	
H16A	0.16900	1.22980	−0.03720	0.0560*	
H1SA	−0.035 (2)	2.289 (9)	−0.284 (3)	0.1210*	0.500
H1O	−0.02100	2.04990	−0.17790	0.0740*	0.500
H1SB	0.0081 (13)	2.124 (4)	−0.2876 (18)	0.1210*	

### Atomic displacement parameters (Å^2^)

**Table:**

	*U*^11^	*U*^22^	*U*^33^	*U*^12^	*U*^13^	*U*^23^
O1	0.0529 (7)	0.0772 (9)	0.0909 (9)	0.0092 (6)	0.0428 (6)	0.0195 (7)
O2	0.0765 (9)	0.0569 (8)	0.0916 (9)	0.0183 (7)	0.0265 (8)	0.0293 (7)
N1	0.0401 (6)	0.0436 (7)	0.0416 (6)	−0.0025 (5)	0.0140 (5)	−0.0025 (5)
N2	0.0390 (6)	0.0523 (8)	0.0480 (7)	−0.0009 (6)	0.0209 (5)	0.0012 (6)
C1	0.0407 (7)	0.0440 (8)	0.0368 (7)	−0.0082 (6)	0.0148 (6)	−0.0020 (6)
C2	0.0462 (8)	0.0579 (10)	0.0513 (8)	−0.0068 (7)	0.0206 (7)	0.0019 (8)
C3	0.0542 (9)	0.0640 (11)	0.0575 (9)	−0.0095 (8)	0.0298 (8)	0.0059 (8)
C4	0.0584 (9)	0.0525 (10)	0.0423 (8)	−0.0109 (8)	0.0207 (7)	0.0026 (7)
C5	0.0480 (8)	0.0463 (9)	0.0346 (7)	−0.0061 (7)	0.0139 (6)	−0.0019 (6)
C6	0.0420 (7)	0.0431 (8)	0.0339 (7)	−0.0057 (6)	0.0137 (5)	−0.0033 (6)
C7	0.0627 (10)	0.0472 (9)	0.0451 (8)	−0.0005 (8)	0.0181 (7)	0.0033 (7)
C8	0.0598 (10)	0.0561 (11)	0.0604 (10)	0.0137 (8)	0.0238 (8)	0.0054 (8)
C9	0.0508 (9)	0.0624 (11)	0.0618 (10)	0.0030 (8)	0.0284 (8)	0.0031 (8)
C10	0.0495 (8)	0.0500 (9)	0.0486 (8)	−0.0012 (7)	0.0235 (7)	0.0034 (7)
C11	0.0420 (7)	0.0430 (8)	0.0392 (7)	−0.0017 (6)	0.0144 (6)	−0.0034 (6)
C12	0.0401 (7)	0.0571 (10)	0.0548 (9)	0.0040 (7)	0.0197 (7)	−0.0062 (8)
C13	0.0443 (8)	0.0492 (10)	0.0638 (10)	0.0121 (7)	0.0124 (7)	−0.0031 (8)
C14	0.0518 (9)	0.0412 (9)	0.0516 (8)	0.0063 (7)	0.0134 (7)	0.0015 (7)
C15	0.0509 (8)	0.0481 (9)	0.0544 (9)	0.0072 (7)	0.0259 (7)	0.0060 (7)
C16	0.0436 (7)	0.0472 (9)	0.0506 (8)	0.0105 (7)	0.0205 (6)	0.0050 (7)
O1S	0.0556 (12)	0.0450 (13)	0.0529 (12)	0.0055 (11)	0.0286 (10)	0.0051 (10)
C1S	0.105 (3)	0.058 (2)	0.090 (2)	0.0000	0.052 (2)	0.0000

### Geometric parameters (Å, º)

**Table:**

O1—C2	1.295 (2)	C11—C16	1.384 (2)
O2—C14	1.362 (2)	C11—C12	1.385 (2)
O2—H2B	0.8200	C12—C13	1.380 (3)
O1S—C1S	1.347 (3)	C13—C14	1.376 (2)
O1S—H1SB^i^	0.76 (3)	C14—C15	1.389 (2)
O1S—H1O	0.9200	C15—C16	1.372 (2)
N1—N2	1.2848 (19)	C3—H3A	0.9300
N1—C1	1.3538 (18)	C4—H4A	0.9300
N2—C11	1.4027 (18)	C7—H7A	0.9300
N2—H2A	0.8600	C8—H8A	0.9300
C1—C6	1.449 (2)	C9—H9A	0.9300
C1—C2	1.429 (2)	C10—H10A	0.9300
C2—C3	1.426 (3)	C12—H12A	0.9300
C3—C4	1.341 (3)	C13—H13A	0.9300
C4—C5	1.428 (2)	C15—H15A	0.9300
C5—C7	1.405 (2)	C16—H16A	0.9300
C5—C6	1.4093 (19)	C1S—H1SA	1.11 (6)
C6—C10	1.402 (2)	C1S—H1SB	0.87 (3)
C7—C8	1.362 (3)	C1S—H1SA^i^	1.11 (6)
C8—C9	1.390 (3)	C1S—H1SB^i^	0.87 (3)
C9—C10	1.370 (3)		
			
C14—O2—H2B	109.00	C4—C3—H3A	119.00
C1S—O1S—H1O	104.00	C2—C3—H3A	119.00
C1S—O1S—H1SB^i^	37 (2)	C3—C4—H4A	119.00
H1O—O1S—H1SB^i^	67.00	C5—C4—H4A	119.00
N2—N1—C1	118.42 (14)	C5—C7—H7A	120.00
N1—N2—C11	119.27 (13)	C8—C7—H7A	120.00
N1—N2—H2A	120.00	C9—C8—H8A	120.00
C11—N2—H2A	120.00	C7—C8—H8A	120.00
C2—C1—C6	120.15 (13)	C8—C9—H9A	120.00
N1—C1—C2	123.90 (14)	C10—C9—H9A	120.00
N1—C1—C6	115.94 (13)	C6—C10—H10A	120.00
O1—C2—C1	121.39 (16)	C9—C10—H10A	120.00
O1—C2—C3	120.43 (16)	C11—C12—H12A	120.00
C1—C2—C3	118.16 (16)	C13—C12—H12A	120.00
C2—C3—C4	121.44 (18)	C12—C13—H13A	120.00
C3—C4—C5	122.32 (16)	C14—C13—H13A	120.00
C4—C5—C7	121.51 (14)	C16—C15—H15A	120.00
C6—C5—C7	119.48 (15)	C14—C15—H15A	120.00
C4—C5—C6	119.01 (14)	C11—C16—H16A	120.00
C1—C6—C5	118.87 (13)	C15—C16—H16A	120.00
C1—C6—C10	122.60 (13)	O1S—C1S—O1S^i^	64.2 (2)
C5—C6—C10	118.53 (14)	O1S—C1S—H1SA	120 (3)
C5—C7—C8	120.54 (16)	O1S—C1S—H1SB	95.7 (18)
C7—C8—C9	120.30 (18)	O1S—C1S—H1SA^i^	121 (3)
C8—C9—C10	120.39 (17)	O1S—C1S—H1SB^i^	31.5 (18)
C6—C10—C9	120.76 (16)	H1SA—C1S—H1SB	106 (3)
N2—C11—C12	117.83 (14)	O1S^i^—C1S—H1SA	121 (3)
N2—C11—C16	122.48 (13)	H1SA—C1S—H1SA^i^	107 (4)
C12—C11—C16	119.70 (13)	H1SA—C1S—H1SB^i^	105 (4)
C11—C12—C13	120.33 (16)	O1S^i^—C1S—H1SB	31.5 (18)
C12—C13—C14	119.86 (16)	H1SA^i^—C1S—H1SB	105 (4)
O2—C14—C13	123.34 (15)	H1SB—C1S—H1SB^i^	127 (3)
C13—C14—C15	119.84 (14)	O1S^i^—C1S—H1SA^i^	120 (3)
O2—C14—C15	116.82 (15)	O1S^i^—C1S—H1SB^i^	95.7 (18)
C14—C15—C16	120.34 (16)	H1SA^i^—C1S—H1SB^i^	106 (3)
C11—C16—C15	119.93 (14)		
			
C1—N1—N2—C11	−179.19 (12)	C7—C5—C6—C1	178.85 (13)
N2—N1—C1—C2	−0.5 (2)	C7—C5—C6—C10	−1.0 (2)
N2—N1—C1—C6	178.51 (12)	C4—C5—C7—C8	−178.29 (15)
N1—N2—C11—C12	−178.98 (13)	C6—C5—C7—C8	0.9 (2)
N1—N2—C11—C16	0.9 (2)	C1—C6—C10—C9	−179.35 (14)
N1—C1—C2—O1	−1.3 (2)	C5—C6—C10—C9	0.5 (2)
N1—C1—C2—C3	177.00 (14)	C5—C7—C8—C9	−0.2 (2)
C6—C1—C2—O1	179.78 (14)	C7—C8—C9—C10	−0.3 (3)
C6—C1—C2—C3	−2.0 (2)	C8—C9—C10—C6	0.2 (2)
N1—C1—C6—C5	−176.08 (12)	N2—C11—C12—C13	−179.50 (14)
N1—C1—C6—C10	3.72 (19)	C16—C11—C12—C13	0.7 (2)
C2—C1—C6—C5	2.97 (19)	N2—C11—C16—C15	179.40 (13)
C2—C1—C6—C10	−177.23 (14)	C12—C11—C16—C15	−0.8 (2)
O1—C2—C3—C4	178.23 (16)	C11—C12—C13—C14	0.2 (2)
C1—C2—C3—C4	0.0 (2)	C12—C13—C14—O2	179.21 (15)
C2—C3—C4—C5	1.1 (3)	C12—C13—C14—C15	−0.9 (2)
C3—C4—C5—C6	0.0 (2)	O2—C14—C15—C16	−179.31 (13)
C3—C4—C5—C7	179.16 (15)	C13—C14—C15—C16	0.8 (2)
C4—C5—C6—C1	−1.97 (19)	C14—C15—C16—C11	0.0 (2)
C4—C5—C6—C10	178.22 (13)		

Symmetry code: (i) −*x*, *y*, −*z*−1/2.

### Hydrogen-bond geometry (Å, º)

Cg2 and Cg3 are the centroids of the C5–C10 and C11–C16 rings, respectively.

**Table:**

*D*—H···*A*	*D*—H	H···*A*	*D*···*A*	*D*—H···*A*
O1*S*—H1*O*···O1^ii^	0.92	1.92	2.832 (3)	171
N2—H2*A*···O1	0.86	1.84	2.540 (2)	137
O2—H2*B*···O1*S*	0.82	2.03	2.841 (3)	172
O2—H2*B*···O1*S*^i^	0.82	1.97	2.690 (3)	146
C1*S*—H1*SA*···*Cg*3^iii^	1.11 (6)	2.58 (5)	3.555 (2)	147 (4)
C7—H7*A*···*Cg*2^iv^	0.93	2.73	3.521 (2)	144

Symmetry codes: (i) −*x*, *y*, −*z*−1/2; (ii) −*x*, −*y*+3, −*z*; (iii) −*x*, *y*+1, −*z*−1/2; (iv) −*x*+1/2, *y*−1/2, −*z*+1/2.
